# Sirtuins and Their Implications in the Physiopathology of Gestational Diabetes Mellitus

**DOI:** 10.3390/ph18010041

**Published:** 2025-01-01

**Authors:** Katarzyna Zgutka, Marta Tkacz, Marta Grabowska, Wioletta Mikołajek-Bedner, Maciej Tarnowski

**Affiliations:** 1 Department of Physiology in Health Sciences, Faculty of Health Sciences, Pomeranian Medical University, 70-210 Szczecin, Poland; katarzyna.zgutka@pum.edu.pl (K.Z.); marta.tkacz@pum.edu.pl (M.T.); 2 Department of Histology and Developmental Biology, Faculty of Health Sciences, Pomeranian Medical University, 71-210 Szczecin, Poland; marta.grabowska@pum.edu.pl; 3 Department of Obstetrics and Gynecology, Pomeranian Medical University in Szczecin, 70-204 Szczecin, Poland; wioletta.mikolajek.bedner@pum.edu.pl

**Keywords:** sirtuins, pregnancy, gestational diabetes, inflammation

## Abstract

Gestational diabetes mellitus (GDM) imposes serious short- and long-term health problems for the mother and her child. An effective therapeutic that can reduce the incidence of GDM and improve long-term outcomes is a major research priority and is very important for public health. Unfortunately, despite numerous studies, the molecular mechanisms underlying GDM are not fully defined and require further study. Chronic low-grade inflammation, oxidative stress, and insulin resistance are central features of pregnancies complicated by GDM. There is evidence of the involvement of sirtuins, which are NAD+-dependent histone deacetylases, in energy metabolism and inflammation. Taking these facts into consideration, the role of sirtuins in the pathomechanism of GDM will be discussed.

## 1. Introduction

Throughout pregnancy, particular maternal adaptations occur in multiple systems, including cardiovascular, respiratory, and metabolic. These changes in maternal physiology aim to maintain a healthy balance between the mother and foetus while ensuring proper foetal development. One of the most critical adaptations is the proper management of energetic substrates in which there is a necessity to shunt glucose to promote foetal development while maintaining adequate maternal nutrition. This regulation is central to maternal–foetal health during all trimesters of gestation.

Glucose utilisation, glycaemia, insulin synthesis, and insulin sensitivity significantly vary throughout pregnancy, constantly adapting to the energy demands of the mother and the foetus. Initially, during gestation, blood glucose levels drop due to the dilutional effect as the maternal blood volume rises, and energetic utilisation further increases during the third trimester of pregnancy [1,2]. To compensate for these changes in maternal insulin sensitivity, hepatic gluconeogenesis and fatty acid levels increase [1]. In the first two trimesters, these metabolic adaptations are aimed at storing essential sources of energy, such as glucose and fatty acids, as fat deposits, which are necessary for the subsequent stages of pregnancy [3]. In parallel, augmented activity of maternal and placenta-derived hormones (oestrogens and progesterone) in the companion of other molecules of placental origin, such as human placental lactogen (hPL) and human placental growth hormone (hPGH), together with changes in the production of inflammatory mediators by the placenta (e.g., tumour necrosis factor α [TNFα]), and cytokines produced by adipose tissue decrease maternal insulin sensitivity. This change results in an elevation of postprandial glucose levels [4] and insulin resistance (IR). Thus, the IR state observed during physiological pregnancy is an adaptive response that favours the rise in blood glucose and free fatty acid levels, shifting energy sources from the mother to the foetus [5,6].

In normal pregnancy, with normal glucose tolerance (NGT), dysglycaemia does not develop because of increased compensatory insulin secretion. It is evidenced by pancreatic hyperplasia and hypertrophy, intensified proliferation, and reduced cell death [7]. IR approximately decreases maternal insulin sensitivity by half, which is balanced by a 250% increase in maternal insulin secretion to maintain normoglycemia [8]. If this compensatory mechanism fails or is too weak, then IR becomes predominant and gestational diabetes mellitus (GDM) develops [2,4,9]. We may conclude that the emerging hyperglycaemia is a result of a deficit in insulin action, resulting in diminished postprandial glucose utilisation by the mother [1]. Other contributing factors may include diminished pancreatic insulin secretion and increased hepatic gluconeogenesis [1,10,11]. GDM arises as the mother’s insulin secretion cannot compensate for the existing hyperglycaemia. Adding to this is a higher level of hepatic gluconeogenesis and significant IR in peripheral tissues [1,4,11,12].

**Figure 1:**
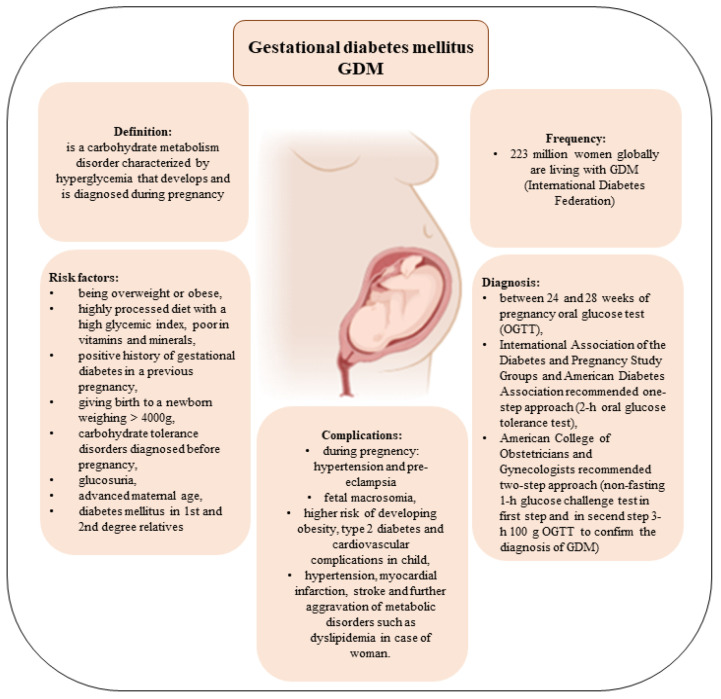
Overview and summary of pathological features, symptoms, and diagnostic strategy of gestational diabetes mellitus.

GDM was first used in 1961 by O’Sullivan [13]. At present, it is the most common metabolic disorder of pregnancy and a serious threat to maternal and neonatal health. Figure 1 summarises the most important facts about GDM.

The prevalence of GDM is increasing rapidly worldwide, in parallel with dramatic changes in lifestyle, the growing incidence of obesity, and the older age of pregnant women. According to the International Diabetes Federation (IDF), more than 200 million women throughout the world are living with GDM [1], and this number is projected to almost double in the next 20 years. It is predicted that one in six births will be affected by GDM [1]. The major contributing factor to GDM and related complications is obesity [14]. A variety of factors elevate the risk of GDM, including excessive weight gain during early adulthood and before 24 weeks of gestation, a history of GDM, advanced maternal age (AMA), a family history of IR and diabetes, obesity (pre-pregnancy body mass index [BMI] ≥ 30 kg/m^2^), pre-pregnancy polycystic ovary syndrome, prior delivery of a newborn more than 4000 g in weight, multi-parity, and ethnicity [1,2,4,9,11,12].

The universal recommendations for GDM testing advise performing screening between 24 and 28 weeks of gestation; testing at this time leads to improved maternal and perinatal outcomes with GDM treatment [15]. However, there is currently no consensus on the optimal diagnostic method for GDM. Two screening approaches (one vs. two steps) are considered acceptable. The one-step approach proposed by the International Association of the Diabetes and Pregnancy Study Groups (IADPSG) and the American Diabetes Association (ADA) exerts a 2 h oral glucose tolerance test (OGTT) in all pregnant women [15,16]. GDM is present if at least one value exceeds the cut-off, including fasting plasma glucose ≥ 92 mg/dL but <126 mg/dL and/or 2 h OGTT ≥ 153 mg/dL. The two-step strategy is recommended by the American College of Obstetricians and Gynecologists (ACOG) and involves a non-fasting 1 h glucose challenge test; it seems to be more comfortable for the participants. If the first step (plasma glucose level ≤ 140 mg/dL) is passed, further screening is usually unnecessary. It is recommended that women who fail the primary screening execute the 3 h 100 g OGTT (>140 mg/dL) to confirm the diagnosis of GDM [15,16].

GDM carries a significant risk of serious health consequences for both the mother and her child. Elevation of the maternal glucose level stimulates foetal hyperglycaemia, which in turn stimulates the release of foetal insulin. As insulin is characterised by both anabolic and metabolic effects, foetal hyperinsulinemia leads to excessive foetal growth and subsequent negative perinatal outcomes, including neonatal hypoglycaemia, shoulder dystocia, prematurity, jaundice, respiratory distress syndrome, and perinatal mortality [17,18,19]. In nulliparous women, significant gestational weight gain in early pregnancy has been associated with the development of GDM and macrosomia [17,18]. Women with GDM usually recover after delivery; however, both the mother and child are more susceptible to type 2 diabetes mellitus (T2DM), obesity, and vascular disorders in the future [11,18,20,21].

## 2. The Pathogenesis of GDM and Its Consequences Involve Multiple Molecular Mechanisms

The background of GDM is composed of diverse genetic variants, dietary factors, and environmental influences. Dysregulation of metabolic pathways, such as glycolysis, β-oxidation, and metabolism of ketone bodies, makes GDM similar in multiple aspects to T2DM [9,13,21]. In addition, the molecular mechanisms and inflammatory pathways involved in T2DM are similarly exploited in GDM and are essential in GDM development, for example, the interleukin 1 beta (IL-1β)/Toll-like receptor 4 (TLR4)/nuclear factor kappa B (NF-κB) pathway.

### 2.1. Insulin Resistance

Insulin resistance (IR) refers to a state in which insulin-sensitive tissues do not respond to insulin adequately. In women with GDM, glucose uptake is almost halved compared with a healthy pregnancy, and IR is enhanced [22]. The major glucose transporter that brings glucose into the cell is insulin-dependent glucose transporter type 4 (GLUT4). The insulin signal is transmitted through insulin receptor substrate (IRS) 1 and finally leads to plasma membrane translocation of GLUT4 [23]. Phosphorylated IRS1 activates two intracellular pathways: the mitogen-activated kinase (MAPK) cascade, which triggers further expression of GLUT1 and GLUT4 [14], and phosphatidyl-inositol 3-kinase (PI3K), which reinforces GLUT translocation to the plasma membrane [24,25]. In IR, insulin signalling is defective due to altered phosphorylation of the insulin receptor or IRS1 and fails to translocate GLUT4 to the cell surface in women with GDM [26]. The current scientific consensus is that pro-inflammatory cytokines contribute to the development of IR [27,28]. Uterine tissues, mostly the placenta, and adipose tissue, are known secretors of pro-inflammatory cytokines, such as TNFα, IL-1β, and IL-6, that impair insulin signalling by inhibiting IRS1 through serine/threonine phosphorylation [29,30].

### 2.2. β-Cell Dysfunction

As mentioned previously, the mechanisms underlying β-cell dysfunction are intricate and diverse and seem to overlap with those present in T2DM. β-cell function is greatly reduced in GDM, and β-cells are unable to compensate for the increase in IR, resulting in the development of GDM [31]. Alterations at various stages of insulin synthesis or secretion have been described, including glucose sensing, pro-insulin synthesis, post-translational modifications, granule storage, and granule exocytosis. Minor deficiencies in β-cell machinery may only become evident during metabolic stress, such as pregnancy. β-Cell dysfunction is triggered by hyperglycaemia and hyperlipidaemia [32]. Interestingly, the majority of susceptibility genes associated with GDM relate to β-cell functions and pancreas development, including the potassium voltage-gated channel KQT-like 1 (KCNQ1), CDK5 regulatory subunit-associated protein 1-like 1 (CDKAL1), melatonin receptor 1B (MTNRB1), and glucokinase (GCK) [6]. Oxidative stress, mitochondrial dysfunction, and endoplasmic reticulum stress result in increasing glucotoxicity and lipotoxicity and deviation of insulin production and β-cell viability [33]. Further release of pro-inflammatory cytokines in a state of chronic inflammation has a detrimental effect on β-cell function via induction of endoplasmic reticulum stress [34,35].

### 2.3. Inflammation Underlying GDM: The NF-κB Signalling Pathway

T2DM involves persistent, chronic, low-grade inflammation. Diverse elements of inflammatory cascade contribute to β-cell dysfunction and IR. The key mediators of inflammation in diabetes include cytokines, adipokines, chemokines, and inflammatory signalling molecules [36]. There is a very important role of the placenta in this myriad of inflammatory elements. As mentioned above, in this specific inflammatory state, the placenta—a transient foetomaternal organ of gestation—is able to produce and respond to various inflammatory stimuli throughout its lifespan.

Cytokines synchronise a paracrine/autocrine communication network operating within the foetomaternal interface to ensure a successful pregnancy. Pro and anti-inflammatory signals are transmitted through the placenta and act on both sides of the foetomaternal barrier [37,38,39]. However, obesity, metabolic diseases, and GDM are associated with altered immune reactions and the formation of a pro-inflammatory environment in adipose tissue, liver, kidney, heart, pancreas, and placenta [4,40,41]. This state of altered, chronic immune responses involves elevation of pro-inflammatory cytokines and chemokines levels (IL-1β, IL-6, and TNFα; and CXCL1, CXCL5, and CXCL8, respectively) and decreased levels of certain anti-inflammatory molecules such as adiponectin, IL-4, and IL-10. From a molecular perspective, the NF-κB signalling pathway plays a central role in eliciting these pro-inflammatory reactions. The proteins in the NF-κB form complexes with stimulative or repressive functions. NF-κB signalling is triggered upon stimulation by the pro-inflammatory cytokines. After receiving signals from the cytokines through their specific receptors or from TLRs, specifically TLR4, the inhibitory regulators of NF-κB are rapidly phosphorylated, ubiquitinated, and then degraded, exposing a nuclear localisation sequence on the NF-κB proteins. Next, the NF-κB dimers affect gene transcription and trigger inflammatory cascades [42,43]. The TLR4/MyD88/NF-κB pathway is active in the placenta and enhances the synthesis and release of inflammatory cytokines and bio-mediators. This, in turn, affects insulin signalling and is conducive to severe insulin resistance in the placentas of women with GDM [44,45]. Of note, TLR4 messenger RNA (mRNA) expression is elevated in the placentas of women with GDM [45].

Inflammation in pregnant women with obesity or GDM can affect foetal development. Clinical studies and research conducted on experimental animal models prove that the inflammatory milieu related to GDM affects the development of neural structures, metabolic pathways and inflammatory responses in offspring [8]. This inflammation has ongoing consequences, including IR, pancreatic dysfunction, oxidative stress, and hyperglycaemia. Hence, there is a need to search for new molecules, agents, or mechanisms that may be utilised for the control of inflammatory states in GDM. One of the most promising and prospective molecular targets in GDM prevention/management is silence information regulators (sirtuins).

## 3. The Roles of Sirtuins in the Pathogenesis of GDM

In this review, we focus our attention on the role of sirtuins in the pathophysiology of GDM. A literature search was performed in widely available databases, such as PubMed, Web of Science, and Google Scholar. We searched for articles using the following keywords and combinations thereof: gestational diabetes mellitus, GDM, diabetes AND sirtuins, SIRT (1–7), hyperglycemia AND sirtuins, inflammation AND GDM, SIRT (1–7) AND chronic inflammation, sirtuins AND insulin resistance (IR). The search was completed in September 2024.

As was mentioned above, IR, inflammation, and abnormal glucose and lipid metabolism play crucial roles in the pathogenesis of GDM. It is well documented that the sirtuin family members act as regulatory factors in pathways associated with these processes [46]. Moreover, sirtuins have a direct role in maintaining glucose homeostasis by controlling the processes of gluconeogenesis and glycolysis, and they also affect insulin secretion [47]. Sirtuins are regulators of metabolism and health span [47]. Seven human sirtuins are known today: SIRT1, SIRT2, SIRT3, SIRT4, SIRT5, SIRT6, and SIRT7 [48].

SIRTs are evolutionarily conserved nicotinamide adenine dinucleotide (NAD+)-dependent deacetylases. Their structures, activities, and locations in the cell are specific and differ between each family member. In general, sirtuins are present in the nucleus (SIRT1, SIRT6, and SIRT7), cytoplasm (SIRT2), and mitochondria (SIRT3, SIRT4, and SIRT5) [48]. However, depending on the physiological/pathophysiological conditions, for example, cellular stress, they might translocate to other organelles to exert their functions [48].

Sirtuins play a significant role in regulating blood glucose levels. They influence various metabolic pathways in the liver, pancreas and skeletal muscle. The balance between these processes enables the proper adjustment of glucose metabolism to the body’s changing energy conditions. In vitro studies have provided strong evidence for the crucial role of sirtuins in glucose homeostasis, making them potential therapeutic targets for the treatment of GDM.

In the sections below, we discuss the general role of the sirtuins in the physiological and pathophysiological control of glucose metabolism and the association between particular SIRTs and diabetes during pregnancy or high glucose in vitro.

### 3.1. SIRT1

SIRT1 is the most well-studied sirtuin family member. It affects multiple biological processes by deacetylating histones and non-histone proteins. SIRT1 is responsible for the deacetylation of lysine 26 of histone H1 (H1K26); lysine 9, lysine 14, lysine 18, and lysine 56 of histone H3 (H3K9, H3K14, H3K18, and H3K56, respectively); and lysine 6, lysine 12, and lysine 16 of histone H4 (H4K6, H4K12, and H4K16, respectively) [49,50]. Deacetylation of promoter-associated H3K9 and H4K16, and subsequent suppression of transcription, is commonly SIRT1-mediated [51]. In addition to histones, mammalian SIRT1 targets numerous other proteins, including a large array of transcription factors, and thus has been implicated in multiple diseases, including diabetes [52]. SIRT1 can directly deacetylate p53, NF-κB, forkhead-box transcription factor 1/3/4 (FOXO1/3/4), heat shock factor 1 (HSF1), hypoxia-inducible factor 1 alpha (HIF-1α), P300, and TIP60 to regulate the transcription of their target genes. Moreover, SIRT1 indirectly promotes the function of transcription factors, such as peroxisome proliferator-activated receptor α/γ (PPARα/γ), myoblast determination protein (MyoD), and others. SIRT1 regulates glucose/lipid metabolism in pancreatic β-cells, positively regulates insulin secretion, and protects cells from oxidative stress and inflammation [52]. The putative regulatory mechanism may work as follows: the induction of insulin secretion is caused by elevated blood glucose levels. After glucose enters pancreatic β-cells, it is metabolised into pyruvate; then, pyruvate enters the mitochondria and produces NADH through the tricarboxylic acid (TCA) cycle, which produces adenosine triphosphate (ATP) through the electron transport chain [53]. SIRT1 represses the expression of uncoupling protein 2 (UCP2) and increases ATP production. Under the condition of food deprivation, pancreatic β-cells downregulate the inhibition of UCP2 by SIRT1 by decreasing the NAD+/NADH ratio and, therefore, increase ATP production and insulin secretion [53]. Moreover, SIRT1 has positive roles in the metabolic pathway via the modulation of insulin signalling by activating the expression of NeuroD and MafA—two transcription factors that regulate insulin expression [49,53]. The role of SIRT1 in the regulation of gluconeogenesis, especially under the condition of caloric restriction (CR), is still debatable [54]. CR induces the expression of SIRT1 in various tissues. Furthermore, CR may influence the upregulation of SIRT1 and PPAR-γ coactivator-1α (PGC-1α) in skeletal muscle. This leads to enhanced mitochondrial function and reduced insulin resistance, metabolic rate, levels of oxidative stress, and visceral fat mass [55]. Liu et al. [56] showed that SIRT1 deacetylates CREB-regulated transcription co-activator 2 (CRTC2), leading to its degradation and decrease of hepatic glucose production. On the other hand, SIRT1 activates FoxO1 and peroxisome proliferator-activated receptor PGC-1γ, hence increasing hepatic glucose production [54]. SIRT1 activates PGC-1α and FoxO1 through a deacetylation reaction and promotes the induction of gluconeogenesis-related genes. Glucose production by the liver is controlled through glucagon-induced cyclic AMP (cAMP) response element-binding (CREB) and CRTC2. This induces the expression of gluconeogenesis-related genes and reduces glucagon’s effects through CRTC2 deacetylation [57]. CR also elevates the expression of SIRT3 in the liver and affects many aspects of the liver’s response to nutrient deprivation [58].

SIRT1 influences insulin pathways by activating insulin receptors and modulating PGC-1α (metabolism-regulating coactivating factor). A diet rich in saturated fats induces IR and reduces PGC-1α expression in muscle tissue. Activation of the SIRT1/PGC-1α signalling pathway has been shown to regulate glucose utilization in skeletal muscle, fasting insulin levels and inhibit IR through activation of the insulin receptor substrate IRS1/PI3K/AKT pathway [59]. Conversely, reduced SIRT1 activity may worsen insulin sensitivity and contribute to the hyperglycemia observed in GDM [49].

Although SIRT1 mostly occur intracellularly, it is released into the bloodstream as a result of cell breakdown or in response to cellular stress/inflammation [60,61]. Wu et al. [62] reviewed the literature published between 2012 and 2022 and found that 74.1% of the included studies assessed sirtuin protein expression in human blood samples (cells and plasma/serum). Several studies have demonstrated the important relationship between SIRT1 and GDM [50,63,64,65]. Ulubasoglu et al. [50] observed that patients with GDM had lower serum SIRT1 levels during the second trimester of pregnancy: 22.0 (19.9–24.3) ng/mL in the GDM group and 34.7 (28.8–54.6) ng/mL in the control/healthy group. The researchers suggested that it may be a diagnostic marker for GDM. On the other hand, Turek et al. [63] observed that SIRT1 mRNA expression in peripheral blood leucocytes was 1.7-fold higher in the GDM group compared with the NGT group, and it correlated positively with the OGTT results in the entire study group and correlated negatively with pregnancy age in the GDM and NGT groups. In addition, there was a positive association between SIRT1 mRNA and the plasma high-density lipoprotein (HDL) cholesterol level in the NGT group. Mac-Marcjanek et al. [65] assessed peripheral blood leucocyte SIRT1 expression at the time of GDM diagnosis. There were 122 patients with GDM divided into two subgroups, namely GDM/SIRT1(↑) and GDM/SIRT1(⇋), with and without significant differences in leucocyte SIRT1 expression compared with the NGT group, respectively. Moreover, the authors identified 11 diabetes-related genes with at least a ±2-fold difference in expression in the GDM/SIRT1(↑) group as compared with the NGT groups. These genes were assigned to five different functional groups: metabolic enzymes (ACLY, G6PD, and GPD1), molecular transport functions (NSF, SNAP23, and STXBP2), transcription factors (PDX1 and SREBF1), signal transduction molecules (IRS1 and IRS2), and an inflammatory factor (IL-6). In addition, the GDM/SIRT1(↑) group was characterised by significantly increased expression of G6PD, IL-6, and SNAP23 and decreased expression of ACLY.

A well-documented, distinct feature of GDM pathophysiology is placental dysfunction [66]. The inflammatory response in the placenta is thought to be a significant causative factor of IR in GDM [67]. Interestingly, Arul Nambi Rajan et al. [68] suggested that SIRT1 is needed for physiological trophoblast differentiation and placental development. In fact, Lappas et al. [64] showed that SIRT1 is expressed in the syncytiotrophoblast layer and the cytotrophoblasts of the placenta, amnion epithelium, trophoblast layer of the chorion, and decidual cells. What is more, SIRT1 expression was significantly lower in placentas from pregnancies complicated with GDM [66]. To shed new light on the pathogenesis of GDM, Han et al. [67] investigated the expression of SIRT1, sterol regulatory element-binding protein-1 (SREBP1), and pyroptosis factors, including NLRP3, caspase-1, IL-1β, and IL-18 in placental tissue and sera of GDM patients (Table 1). SREBP1 is a transcription factor primarily responsible for regulating the synthesis of lipids, such as fatty acids and total cholesterol; moreover, it is a key player in insulin signalling pathways [67]. Pyroptosis is a form of caspase-1-dependent programmed cell death directly related to inflammation, which can be activated by an overabundance of nutrients like glucose and lipids. The authors evaluated a total of 100 pregnant women (50 with GDM and 50 with normal pregnancies). The results showed that there was significantly lower SIRT1 expression in placental tissue and sera from the GDM group compared with the normal pregnancy group. Meanwhile, SREBP1 expression was significantly increased in the GDM group compared with the normal pregnancy group. The serum levels of pyroptosis markers were significantly elevated in the GDM group compared with the normal pregnancy group. There were negative correlations between SIRT1 expression and SREBP1, IL-1β, and IL-18 expression. The authors confirmed that all these factors interact with each other in GDM to form a complex regulatory network involved in the genesis and development of the disease. Zhang et al. [69] demonstrated that SIRT1 is a target for microRNA (miR)-135a5p delivered by the placental exosome. Exosomal miR-135a5p contributes to inflammation, adipogenesis, and glucose metabolism, and, interestingly, its level was elevated in the plasma of women with GDM compared with healthy pregnant women. miR-135a5p delivered by placental exosomes targeted SIRT1 and disrupted GDM-induced cellular dysfunction by activating the PI3K/AKT signalling pathway.

Mishra et al. [70] used syncytialised primary human trophoblasts, treated with or without glucose and insulin for 72 h, to mimic the IR conditions of GDM pregnancies. Then, they assessed the role of SIRT1 in the regulation of docosahexaenoic acid (DHA) transfer across trophoblasts. The GDM conditions significantly suppressed the translocation of DHA transfer, but triglyceride accumulation and fatty acid transporter expression (CD36, FABP3, and FABP4) were increased (Table 1). Moreover, exposure to GDM significantly reduced SIRT1 mRNA and protein expression. The application of SIRT1 inhibitors led to diminished DHA transfer in healthy trophoblasts. Recombinant SIRT1 and SIRT1 activators restored the decreased DHA transport induced by the GDM conditions [70].

Diabetes during pregnancy is associated with adverse pregnancy outcomes. Endothelial cells regulate vascular tone and are the first foetal cells exposed to maternal hyperglycaemia. SIRT1 inhibition in hyperglycaemic conditions results in endothelial cell dysfunction, while SIRT1 activation alleviates endothelial ageing induced by oxidative stress [71]. In pregnancies complicated by GDM, Lappas et al. [64] observed a reduction in SIRT1 expression and activity in foetal endothelial colony-forming cells (ECFCs) and human umbilical vein endothelial cells (HUVECs). Alqudah et al. [72] obtained similar results when they investigated the role of FK506-binding protein like (FKBPL) and SIRT1 in angiogenesis complicated by GDM. What is interesting, SIRT1 reduces oxidative stress and inflammatory reactions by affecting endothelial functions through multiple signalling cascades dependent on adenosine monophosphate-activated protein kinase (AMPK), NADPH oxidases (NOXs), endothelial nitric oxide synthase (eNOS), and FoxOs [71]. It is evident that there is a complex network of interactions between these pathways and SIRT1 [66,71]. A reduction in SIRT1 in foetal cells may potentially link the development of long-term cardiovascular complications in the offspring of women with GDM. However, the mechanisms leading to these pregnancy complications are still poorly understood.

### 3.2. SIRT2

SIRT2 is located mainly in the cytoplasm and is the least understood of the seven mammalian sirtuin isoforms. Previous experiments have shown that SIRT2 is an important player in the maintenance of metabolic homeostasis, inflammatory responses, oxidative stress, and mitochondrial function, as well as adipocyte differentiation, fatty acid oxidation, gluconeogenesis, and insulin sensitivity [73]. Although SIRT2 is primarily located in the cytoplasm, it is also found in the mitochondria, cell membranes, and cytoskeleton in different, metabolically relevant organs such as brain, heart, kidney, liver, pancreas, muscle, and adipose tissue [73,74]. SIRT2 is implicated in the deacetylation of some transcription factors, including p53, NF-κB, and P300 [74]. Moreover, other proteins involved in cellular metabolism and oxidative stress are deacetylated and modulated by SIRT2. Similarly to SIRT1, SIRT2 regulates inflammation by deacetylating lysine 310 of the p65 subunit of NF-κB, which results in decreased expression of NF-κB [73,75]. Whether this deacetylation promotes or inhibits the expression of pro-inflammatory genes is still unclear. The final results of SIRT2 action on inflammation vary depending on the inflammatory stimuli, model, the type of cells, and the context, making it difficult to conclude whether SIRT2 could have a pro- or anti-inflammatory function [74]. Suppression of excessive hepatic gluconeogenesis is an effective strategy for controlling hyperglycaemia in T2DM. Moreover, SIRT2 is a critical sensor in cellular glucose levels by deacetylating and stabilising phosphoenolpyruvate carboxykinase 1 (PEPCK1), an important enzyme in gluconeogenesis [53,76]. Upon activation of SIRT2 by hypoglycaemia, PEPCK1 is stabilised and shifts the equilibrium towards the generation of glucose from non-carbohydrate carbon sources. On the contrary, when high glucose concentration is present, SIRT2 expression is suppressed, leading to PEPCK1 degradation via ubiquitination [76]. Ren et al. [77] demonstrated that inhibition of cytoplasmic Sirtuin activity (mainly SIRT2 activity) in primary mouse hepatocytes played a crucial role in regulating the PEPCK1 acetylation level. It was reported that the SIRT2 inhibitor, sirtinol, promoted PEPCK1 acetylation and degradation, thereby decreasing cellular gluconeogenesis [77]. SIRT2 also acts as a key component of a signalling network required to maintain the status of IR, but its role is controversial [54]. Recently, SIRT2 was described as a novel AKT interactor critical for AKT activation by insulin [78]. Inhibition of SIRT2 in preadipocytes decreases AKT activation, while its overexpression enhances the activation of AKT and its downstream targets, such as glycogen synthase kinase 3 (GSK3) and p70-S6-kinase. On the other hand, Arora et al. [79] reported that SIRT2 is upregulated in insulin-resistant skeletal muscle cells. Pharmacological or genetic inhibition of SIRT2 improves the phosphorylation of AKT and GSK3β, thus increasing insulin-stimulated glucose uptake. These bilateral roles of SIRT2 need further investigation. Zhang et al. [80] analysed the expression of PI3K, protein kinase B (PKB), and GSK3β in the skeletal muscles of patients with GDM. They observed that the serine phosphorylation levels of PKB and GSK3β were significantly lower in the GDM group compared with the control group. The authors concluded that the downregulation of PI3K, PKB, and GSK3β in the skeletal tissue of patients with GDM is caused by dysfunctional phosphorylation of signalling molecules, leading to IR and glucose transporter functional decline (Table 1).

SIRT2 also contributes to the regulation of insulin sensitivity. Belman et al. [81] identified a tether containing a UBX domain for GLUT4 (TUG) as a novel target for SIRT2. They reported that SIRT2 binds to and deacetylates TUG. The authors proposed TUG as the first molecular marker for glucose transporter 4 storage vesicles (GSVs) and that it regulates the basal intracellular retention and insulin-stimulated release of these vesicles. Overexpression of SIRT2 reduces TUG acetylation and redistributes GLUT4 and insulin-regulated aminopeptidase (IRAP) to the plasma membrane in adipocytes [81].

Little is known about the function of SIRT2 in the context of pregnancy and GDM. Regarding the localisation, it has been elucidated that SIRT2 is highly expressed in decidual cells, cytotrophoblasts, syncytiotrophoblasts, amniotic epithelial cells, chorionic trophoblast cell layer, and in the placental endothelium [48]. During physiological pregnancy, the mean ± standard deviation SIRT2 level is 1.7 ± 1.0 ng/mL in maternal serum and 4.5 ± 3.13 ng/mL in the foetal cord serum [61]. Unfortunately, there are no data on how the levels change during pregnancy complicated by GDM.

Yang et al. [82] found that maternal diabetes in vivo and high glucose in vitro significantly reduces SIRT2 expression through oxidative stress. SIRT2 downregulation-induced histone acetylation may be involved in diabetes-induced neural tube defects (NTDs). However, additional experiments are still needed to fill the gap in knowledge about the role of this sirtuin in the molecular mechanism of GDM.

### 3.3. SIRT3

SIRT3 has wide enzymatic activity. Full-length SIRT3 (44 kDa) resides in the nucleus and exerts epigenetic control by deacetylating histones [83]. When SIRT3 enters the mitochondria, it is cleaved at its N-terminus by matrix-processing peptidase to become a 28-kDa enzymatically active mature protein. SIRT3 is a prototypical NAD+-dependent mitochondrial deacetylase and is responsible for the bulk of mitochondrial protein deacetylation [84].

SIRT3 regulates several mitochondrial functions, including ATP generation, nutrient oxidation, and cell death. It has an important role in maintaining homeostasis, particularly under stress conditions [84,85]. SIRT3 also plays a key role in regulating mitochondrial reactive oxygen species (ROS) homeostasis. Increased ROS production in vascular tissue under pathological conditions is the main cause of endothelial dysfunction [86]. SIRT3 is important in the inhibition of ROS production. Guo et al. [58] demonstrated that in obese mice fed a high-fat diet, hepatic gluconeogenesis was reduced by decreasing enzymatic activities of glucose-6-phosphatase and fructose-1,6-bisphosphatase (PEPCK), pyruvate carboxylase (PC), and malate dehydrogenase 2 (MDH2) through the downregulation of SIRT3 expression [58]. SIRT3 has beneficial effects on glucose metabolism by increasing insulin sensitivity and decreasing serum glucose levels [87]. It is also involved in skeletal muscle metabolism. The deletion of SIRT3 in vivo induces hyperacetylation of the pyruvate dehydrogenase (PDH) E1α subunit, leading to decreased enzymatic activity. As a result, glucose oxidation is reduced, causing a metabolic switch to fatty acid β-oxidation. This leads to loss of skeletal muscle metabolic flexibility and increased insulin resistance [88]. These findings indicate the role of SIRT3 deficiency in the pathogenesis of IR and T2DM. Moreover, SIRT3 protects β-cells from cell death or damage under oxidative stress. Loos of SIRT3 sensitises cells to apoptosis and blocks the protective effect of NAD+ due to the harmful accumulation of intracellular ROS [89].

Maternal obesity significantly reduces placental SIRT3 levels [90]. Sonkar et al. [91] investigated the effect of perfluorooctanesulfonic acid (PFOS) on ROS generation in a first-trimester human trophoblast cell line (HTR-8/SVneo). This global pollutant significantly increased ROS production and decreased the expression of SIRT3, which may lead to pregnancy complications. SIRT3, through its interaction with PGC1α, can increase respiratory capacity and reduce oxidative stress by attenuating mitochondrial ROS generation [62,86,92]. However, increased ROS production is associated with increased oxygen consumption and hyperglycaemia [86]. PGC1α integrates different cellular signals and regulates genes involved in energy metabolism, such as SIRT3. In turn, PGC1α-dependent mitochondrial metabolic reprogramming mediated by SIRT3 could be one of the reasons for altered metabolic regulation of GDM cells later on in children of GDM pregnancies [86]. Moreover, SIRT3 participates in regulating the phosphorylation of AMPK, is related to the maintenance of cellular redox balance, and promotes autophagy [85]. In vitro studies using the HTR8/SVneo cell line showed significantly elevated SIRT3 protein expression in the cells exposed to high glucose concentrations. Increased SIRT3 expression contributes to classical ferroptotic events and autophagy activation by promoting the AMPK/mammalian target of the rapamycin complex (mTOR) pathway and decreasing glutathione peroxidase 4 (GPX4) levels, while SIRT3 silencing leads to resistance against both ferroptosis and autophagy (Table 1).

Gui et al. [86] demonstrated that foetal ECFCs and HUVECs from pregnancies complicated by GDM have decreased NAD+ concentrations. However, only ECFCs showed reduced SIRT3 activity and lower SIRT3 transcript levels, which could potentially lead to the development of long-term cardiovascular complications in the offspring of GDM pregnancies.

SIRT3 contributes to the deacetylation of up to 60% of mitochondrial proteins involved in oxidative phosphorylation, glycolysis, and fatty acid oxidation [83]. Furthermore, SIRT3 participates in glucose metabolism through negative regulation of aerobic glycolysis by inhibiting HIF-1α [62] and promotes the incorporation of amino acids into gluconeogenesis by activating glutamate dehydrogenase (GDH) activity and simultaneously inhibiting glycolysis. SIRT3 regulates high glucose-induced oxidative stress through the deacetylation and activation of superoxide dismutase 2 (SOD2), and the activity of this enzyme is inhibited when the SIRT3 gene is deleted [83,92]. SIRT3 gene knockdown increases apoptosis and cellular ROS in pancreatic islet β-cells isolated from patients with T2DM [93]. In turn, SIRT3 mRNA and manganese superoxide dismutase (MnSOD) enzyme activity are lower in the skeletal muscle of women with obesity and NGT or GDM compared with pregnant women with normal weight [93,94]. Data from postpartum women with previous GDM showed lower SIRT3 and SOD2 expression levels relative to the controls [92].

SIRT3 negatively regulates pro-inflammatory cytokines (TNFα, IL-1β, and IL-6) by modulating the NF-κB signalling pathway [62]. Lim et al. [95] reported that SIRT3 is involved in regulating the mediators of human labour and delivery. The pro-inflammatory cytokines IL-1β and TNFα, capable of inducing uterine contractions, significantly decrease SIRT3 expression in the myometrium. Whether the decrease in SIRT3 expression in human term labouring myometrium is a cause or consequence of human labour is not known. However, the decrease may be responsible for the increased inflammation associated with the onset of labour termination.

In addition to its involvement in the pathogenesis of GDM, SIRT3 is also associated with the development of T2DM in women with GDM and their offspring. SIRT3 activity is essential for the proper functioning of the pancreas and the regulation of insulin signalling pathways through regulating oxidative stress. SIRT3 deacetylates and activates components of the electron transport chain complexes, thereby reducing ROS production [96]. Caton et al. [93] reported that SIRT3 plays a key role in the regulation of pancreatic β-cell function and in their protection from apoptotic cell death. Moreover, lower levels of SIRT3 are associated with increased levels of ROS in cells that can have toxic effects on β-cells. It seems, therefore, that loss of SIRT3-mediated regulation of ROS production could be a mechanism driving the abnormal activity of β-cell. [93,96]. On the other hand, SIRT3 overexpression inhibits ER stress and attenuates palmitate-induced pancreatic β-cell dysfunction [54]. Increasing the activity of this enzyme could be used for therapeutic purposes in patients with GDM and T2DM [90,93].

### 3.4. SIRT4

SIRT4 is localised primarily in mitochondria and has a mitochondrial targeting sequence [62]. It can catalyse NAD+-dependent deacylation (deacetylation, demalonylation, and desuccinylation) and ADP-ribosylation that modulate the mitochondrial metabolic function in the cells [89]. SIRT4 is an important regulator of lipid homeostasis and is a cellular, especially mitochondrial, lipoamidase. One of its targets is pyruvate dehydrogenase, which is inhibited following SIRT4-dependent lipoamide hydrolysis. This may promote the maintenance of pyruvate dehydrogenase activity, thereby enabling glucose metabolism [86]. Several SIRT4 activities may contribute to the regulation of insulin secretion in pancreatic β-cells [97]. For example, its ADP-ribose transferase activity can downregulate GDH activity in β-cells, and it may play a role in regulating amino acid-stimulated insulin secretion. Moreover, the regulation of leucine catabolism and adenine nucleotide translocase 2 (ANT2) activity by SIRT4 may contribute to the ability of SIRT4 to regulate insulin secretion. This process promotes decreasing the level of ATP in the cell and reducing the insulin secretion response [62,97,98]. SIRT4 inhibits insulin secretion as an ADP-ribosyltransferase in pancreatic β-cells and regulates insulin sensitivity [99]. Overall, SIRT4 functions as a negative regulator of insulin secretion in β-cells [100]. However, during fasting, SIRT4 activity is inhibited, which promotes gluconeogenesis from amino acids and fats and enables insulin secretion from β-cells [87].

Deacylase methylcrotonyl-CoA carboxylase (MCCC) regulates branched-chain amino acid (BCAA) catabolism in cells, and specifically functions in leucine catabolism [97]. There are elevated plasma levels of amino acids, especially BCAAs, in women with GDM. Nevertheless, the complex association between diabetes and BCAAs is not fully understood [101]. One of the possible mechanisms assumes that increased levels of BCAAs activate mTORC1, which stimulates the phosphorylation of IRS1 and the insulin receptor [102]. The second possible mechanism could be related to the accumulation of potentially toxic BCAA metabolites induced by modified BCAA metabolism. It results in the induction of oxidative stress, mitochondrial dysfunction, impaired insulin action, and altered glucose homeostasis [102]. In turn, SIRT4 promotes leucine catabolism via MCCC activation, which could modulate glutamine-stimulated insulin secretion by decreasing the concentration of leucine and preventing GDH activation [97].

SIRT4 represses fatty acid oxidation while promoting lipid anabolism by inhibiting malonyl CoA decarboxylase (MCD), an enzyme that produces acetyl CoA from malonyl CoA [103]. SIRT4 also inhibits the activity of the pyruvate dehydrogenase complex (PDH), which catalyses the conversion of pyruvate into acetyl-CoA [104].

Decreased SIRT4 expression reduces the proliferation and invasive capacity of trophoblasts and may induce intracellular oxidative stress and inflammatory responses [60]. SIRT4 deficiency activates the phosphorylation of NF-κB in endothelial cells [105,106]. Silencing SIRT4 increases the expression of pro-inflammatory cytokines (IL-1β, IL-6, and IL-8), cyclooxygenase-2 (COX2), matrix metalloproteinase-9 (MMP9), and intercellular adhesion molecule-1 (ICAM-1) in HUVECs [106]. In turn, SIRT4 overexpression significantly attenuates the inflammatory response [107]. Both ECFCs and HUVECs from GDM pregnancies show lower SIRT4 transcript levels [86]. These data may provide insight into the potential mechanisms of the pathophysiology of long-term cardiovascular complications observed in the offspring of women with GDM. On the other hand, Maghbooli et al. [108] speculated that increased expression of SIRT4 in peripheral blood mononuclear cells (PBMCs) from patients with T2DM and retinopathy can be interpreted as protective feedback in PBMCs to counteract hyperglycaemic and oxidative milieu in retinopathy.

It is worth mentioning that the levels and activity of SIRT4 have been implicated in modulating susceptibility to hyperinsulinemia and diabetes [97]. There are limited data on the role of SIRT4 in regulating inflammation and IR associated with GDM. Nevertheless, it appears that the molecular mechanisms in T2DM are similar, and it is assumed that SIRT4 downregulation is likely to accelerate the development of GDM [86].

### 3.5. SIRT5

SIRT5 is mainly involved in the regulation of mitochondrial metabolism. However, it is worth noting that a significant amount of SIRT5 has also been found in the cytosol in human 293T cells [109,110]. SIRT5 participates in regulating processes such as cellular redox and ROS homeostasis [111], energetic flux through glycolysis [112], amino acid degradation, and fatty acid oxidation [109]. Additionally, studies conducted on the MDA-MB-231 human breast carcinoma cell line suggest that SIRT5 is involved in the regulation of ammonia production and autophagy, which is induced by ammonia, by regulating the metabolism of glutamine [113]. Phylogenetic analysis showed that SIRT5 is distinct from other mammalian sirtuins. Based on the analysis of the aligned conserved core domains of sirtuins, SIRT5 has been assigned to the third class of sirtuins [114]. In vitro and in vivo studies have revealed that mitochondrial SIRT5 is a NAD+-dependent protein lysine demalonylase, desuccinylase, and deglutarylase. Moreover, SIRT5 exhibits weak deacetylating activity compared with SIRT1 and SIRT3. Of note, the catalytic efficiencies for demalonylation, desuccinylation, and deglutarylation of lysine residues of target proteins are much stronger than that for deacetylation [109,115,116]. SIRT5 is involved in the regulation of many cellular pathways, and disturbances in its activity may be associated with the development of various diseases, including tumour development, metabolic disorders, neurodegenerative diseases, cardiovascular pathologies, and infectious diseases [110,113,117]. Unfortunately, there have been no scientific reports regarding the role of SIRT5 in the pathomechanism of GDM. Data on the role of SIRT5 independently in T2DM are also very limited. Ma et al. [118] reported increased SIRT5 activity in patients with T2DM. The authors also found a positive association between elevated SIRT5 expression and age and blood glucose levels and negative associations with pancreatic and duodenal homeobox 1 (PDX1) expression, which is the earliest tissue-selective transcription factor expressed in the developing primordium and is essential for the formation of all pancreatic cell types and the activity of adult islet β-cells. Moreover, other studies have shown that SIRT5 is involved in ketone body synthesis, the TCA cycle, β-oxidation of fatty acids, amino acid catabolism, glycolysis, and ATP synthesis [119]. Recently, an experiment in two pancreatic β-cell lines, MIN6 and INS, showed that inhibition of SIRT5 promotes proliferation of pancreatic β-cells and insulin secretion [118]. This study implies that SIRT5 can promote the progression of T2DM.

The specific environment within the ovarian follicle plays an important role in the processes of support and development of a competent oocyte. Proper bidirectional communication between the oocyte and the surrounding granulosa and cumulus cells is crucial for, among others, the establishment of a viable pregnancy [120,121]. Pacella-Ince et al. [121] found the presence of SIRT5 within human ovarian follicular cells and suggested that reduced SIRT5 gene expression may be associated with decreased oocyte quality in women with reduced ovarian reserve or AMA. In turn, decreased SIRT5 expression may lead to reduced activity of carbamoyl phosphate synthase 1 (CPS1), a target of SIRT5, and consequently to the accumulation of ammonia in the follicular fluid (Table 1). Vaigauskaitė et al. [122] suggested the importance of follicular fluid, secreted by granulosa cells in women, as an indicator for GDM.

Interestingly, oocyte donation has recently been speculated as an alternative for women with poor oocyte quality; however, the studies performed in women after in vitro fertilisation with oocyte donation revealed an increased risk for GDM in pregnancies [123]. By contrast, Akamine et al. [124] found that embryo quality is not associated with GDM. The studies have also shown that AMA is a key risk factor for GDM and adverse pregnancy outcomes, including preterm birth and pre-eclampsia [125]. Hence, it can be assumed that AMA is the cause of many adverse changes, including the decrease in SIRT5. However, there is no evidence of a direct relationship between SIRT5, oocyte quality, and GDM.

Ren et al. [126] revealed that SIRT5 overexpression could decrease the level of protein kinase AMP-activated catalytic subunit alpha 2 (PRKAA2) succinylation in primary placental cells isolated from the placental tissues of patients with hypertensive disorder complicating pregnancy (HDCP). Moreover, the authors showed that PRKAA2 is upregulated in patients with HDCP, and SIRT5 regulates primary placental cell apoptosis through PRKAA2. They also demonstrated a direct interaction between SIRT5 and PRKAA2. Interestingly, in the placentas of women with obesity and GDM, researchers noted PRKAA2 downregulation [127]. Therefore, it can be speculated that SIRT5 levels will be reduced in patients with GDM.

Nishida et al. [112] found that glycolysis and gluconeogenesis are the main pathways regulated by SIRT5. In primary hepatocytes from mice without SIRT5, glycolytic flux was diminished. In glyceraldehyde 3-phosphate dehydrogenase (GAPDH), which is a SIRT5 demalonylation substrate, the substitution of lysine residue 184 with a malonyllysine mimic inhibits its enzymatic activity. The results indicate that SIRT5 may contribute to the regulation of glucose metabolism and insulin response. Jukarainen et al. [128] conducted a study on young adult monozygotic twin pairs and observed that in the heavier co-twins of the BMI-discordant twin pairs, SIRT5 expression was significantly downregulated. SIRT5 correlated negatively with the adiposity variables (including BMI, body fat percentage, subcutaneous adipose tissue volume, intra-abdominal fat volume, liver fat percentage, plasma leptin, and the average adipocyte diameter) and correlated positively with measures of insulin sensitivity variables (including the homeostatic model of assessment of insulin resistance [HOMA-IR], insulin receptor [INSR] gene expression, and IRS2 gene expression).

### 3.6. SIRT6

Similar to SIRT1, SIRT6 is located mainly in the nucleus; however, its sub-nuclear localisation may change during the cell cycle. During the S phase of the cell cycle, SIRT6 is present in heterochromatic regions and is excluded from the nucleolus, but during the G1 phase, it is detected in the nucleolus. During the rest of the interphase, SIRT6 can also be observed in the nucleolus, and when overexpressed, it causes a slowdown in mitosis [129].

Together with SIRT1, SIRT6 is a negative regulator of NF-κB activity [130]. It also shows mono-ADP-ribosyltransferase (including autoribosylation, as well as ribosylation of Kap1 and PARP1 in response to various cellular stresses) and deacylase activities and can catalyse long-chain fatty deacylation [131].

SIRT6 is expressed in the chorionic trophoblasts of the foetal membranes, in the amnion epithelial cells, and in decidua [132]. It is necessary for genomic stability, telomeric maintenance, and recruitment of a chromatin remodeller in DNA damage response [131,133]. It also plays an important role in tumour suppression [134] and in the regulation of glucose homeostasis in the whole body and in local tissues such as the liver and skeletal muscle [62].

SIRT6 improves glucose metabolism by regulating glycolysis and gluconeogenesis, affecting insulin secretion and signalling [89]. During gluconeogenesis, SIRT6 is modulated by factors such as the PGC-1α and FoxO1. Notably, SIRT6 overexpression enhances the utilization of two major gluconeogenic precursors (glycerol and lactate) while preventing age-dependent deterioration of euglycemia and gluconeogenic capacity [135]. Furthermore, SIRT6 negatively regulates HIF-1α to control glycolysis-related pathways, such as pyruvate dehydrogenase kinase (PDK). PDK increases the conversion of glucose to pyruvate and lactate, facilitating adaptation to hypoxia [136]. In a T2DM mouse model, SIRT6 inhibition has been shown to increase the expression of glucose transporters and glycolytic enzymes, thereby reducing blood glucose levels [136]. SIRT6 also plays an important role in the regulation of insulin secretion. In beta cell-specific Sirt6 knockout mice, glucose-stimulated insulin secretion was reduced by 50% [137]. Additionally, SIRT6 deficiency leads to abnormal upregulation of thioredoxin-interacting protein in β-cells, which further impairs insulin secretion. Moreover, SIRT6 regulates glucose-stimulated insulin secretion by modulating mitochondrial glucose oxidation, plasma membrane depolarization, and calcium dynamics [137].

For example, in pancreatic β-cells, SIRT6 deacetylates FoxO1 and subsequently increases the expression of glucose-dependent transporter 2 to maintain the glucose-sensing ability of pancreatic β-cells and systemic glucose tolerance [62]. There is limited research on the relationship between SIRT6 protein and T2DM. Nevertheless, SIRT6 is considered to be a potential biomarker in the pathomechanism of T2DM glycolipid metabolism. Bian et al. [138] revealed that SIRT6 expression in the sera of patients with T2DM increases with the severity of this disease.

In this context, attention has been paid to the participation of SIRT6 in the regulation of IR, obesity, and energy metabolism [139]. However, there are limited data available regarding the potential involvement of SIRT6 in the pathogenesis of GDM. Recent studies have indicated many important functions of SIRT6 during pregnancy by regulating foetomaternal inflammation and cell differentiation [132,140]. Ferrer et al. [140] reported that homozygous inactivating mutation in the SIRT6 gene (the Asp63His [D63H] mutation) in humans is associated with perinatal lethality and multiple prenatal abnormalities, including microcephaly, intrauterine growth restriction, sex reversal in male foetuses, cephalic and craniofacial foetal abnormalities, and congenital heart defects. In vitro studies performed as part of this research revealed that SIRT6 activity is essential to directly silence core pluripotent gene expression (Oct4, Nanog, and Sox2). In effect, SIRT6-deficient human-induced pluripotent stem cells (iPSCs) derived from D63H homozygous foetuses fail to differentiate into functional cardiomyocyte foci, embryoid bodies, and neural progenitor cells due to the lack of repression of pluripotent genes.

Lim et al. [132] found that SIRT6 regulates pro-labour mediators in human foetal membranes. The authors suggested that mediators of preterm labour downregulate SIRT6 expression in foetal membranes, leading to increased activity of NF-κB and its targets. Consequently, this may lead to the premature rupture of membranes and preterm birth. SIRT6 knockdown in primary amnion cells by siRNA is associated with increased production and release of proinflammatory cytokines and mediators. Moreover, lipopolysaccharide (LPS), one of the components of the outer wall of Gram-negative bacteria, decreases SIRT6 mRNA and protein expression [132]. Chronic low-grade inflammation (LGI) promotes IR and affects foetal development. Nguyen et al. [66] showed that in women with GDM in their third trimester of pregnancy, LGI is associated with glucose resistance and IR. Moreover, SIRT6 induces PGC-1α acetylation and suppresses hepatic glucose production. SIRT6 also cooperates with p53 to deacetylate FoxO1 and transport FoxO1 from the nucleus to the cytosol; it also suppresses the expression of gluconeogenic genes. These actions could alleviate diabetic hyperglycaemia [62].

It is worth noting that a number of studies on animal models indicate decreased SIRT6 activity in the context of obesity and diabetes [141,142,143]. Yu et al. [143] found that maternal diabetes modelled in vivo in mice or in vitro based on high glucose conditions suppresses SIRT6 expression through oxidative stress, and histone acetylation induced by sirtuin downregulation may be associated with maternal diabetes-induced NTDs (Table 1). SIRT6 expression increases upon nutrient deprivation in human embryonic kidney HEK293 cells, in mice after fasting, and in rats following a calorie-restricted diet [141]. It is worth noting that SIRT6 deficiency in mice is associated with premature ageing and metabolic disorders. SIRT6 overexpression is related to the downregulation of a selective set of PPAR-responsive genes, as well as genes associated with lipid storage. This may explain the protective role of SIRT6 in the case of metabolic consequences of diet-induced obesity [142].

### 3.7. SIRT7

SIRT7 is located in the nucleus; it is present in the nucleoplasm and is mostly expressed in the nucleolar organiser regions. However, it can also be detected in the cytoplasm [144]. It is involved in deacetylation, desuccinylation, and deglutarylation reactions [145]. SIRT7 seems to exhibit histone H3K18-selective deacetylation activity, although it has numerous other target proteins and shows ADP-ribosyltransferase activity [144,146,147]. SIRT7 is found in all organs and tissues, including the placental tissues and the maternal and foetal cord, with the exception of skeletal muscle [147,148,149]. SIRT7 is associated with the regulation of many cellular processes, including genome stability and gene expression. It plays a key role in activating RNA polymerase I transcription, especially in proliferating cells [150]. According to a recent study, SIRT7 is also involved in the stress response [151] and the regulation of metabolism. There are a few reports indicating the role of this protein in lipid and glucose metabolism [144,152,153]. SIRT7 is involved in glucose metabolism through the acetylation of phosphoglycerate kinase 1 (PGK1), the reduction of which inhibits glycolysis. It has been reported that glucose deprivation stimulates SIRT7 binding to the hepatic glucose-6-phosphatase catalytic subunit (G6PC), leading to elevated G6PC expression and promotion of hepatic gluconeogenesis [54].

SIRT7 destabilises circadian protein cryptochrome 1 (CRY1) and thus inhibits the CRY1-mediated suppression of gluconeogenesis [144]. SIRT7 is also reported to increase the transcription of G6pc through the deacetylation of H3K18 in the G6pc promoter. Moreover, SIRT7 has been implicated in the regulation of glycolysis. Its overexpression decreases HIF-1 protein expression levels, and the effect is independent of its own enzymatic activity. SIRT7 reduces the activity of PGK1 through its deacetylation [144]. Recently, researchers noted that under high glucose conditions, SIRT7 is methylated at arginine 388 (R388), which inhibits the H3K18-deacetylase activity of SIRT7 and stimulates mitochondrial biogenesis [144].

SIRT7 deficiency seems to be involved in ameliorating inflammatory responses. Wyman et al. [154] observed that silencing SIRT7 in the pulmonary artery or microvascular endothelial cells was associated with attenuation of LPS-induced increases in endothelial adhesion molecules (ICAM1 and VCAM-1) and also IL-8 and IL-6. In turn, the loss of endothelial adhesion molecules is accompanied by increased endothelial barrier permeability. These data suggest that SIRT7 may control inflammation. In turn, inflammation promotes IR and may be associated with the development of GDM [155].

Accumulation of fat is associated with the induction of low-grade chronic inflammation via the production of proinflammatory cytokines in adipose tissue. In turn, obesity-induced inflammation may cause IR [156,157]. Kurylowicz et al. [158] found obesity-associated upregulation of SIRT7 mRNA in visceral adipose tissue (VAT) and subcutaneous adipose tissue derived from obese patients. This upregulation is not closely linked with the methylation status of promoters.

Other studies have revealed that SIRT7 negatively regulates the levels of HIF-1α and HIF-2α via a mechanism that is independent of prolyl hydroxylation in cultured cells (HeLa, Hep3B, MDA-MB-231 human breast cancer, and human embryonic kidney 293T cells) [159]. Li et al. [160] demonstrated that HIF-1α expression increases in the GDM human uterus and human uterine smooth muscle cells (HUSMCs) cultured under high glucose and immediate hypoxia conditions. Of note, the authors found that hypoxia is associated with the regulation of uterine smooth muscle contraction and is an important element enabling hyperglycaemia to regulate the process of uterine smooth muscle contraction in patients with GDM.

Some authors have reported that SIRT7 promotes lipogenesis by inducing the methylation of SREBP1a in tumours. The symmetric dimethylation of SREBP1a increases cholesterol, fatty acid, and triglyceride biogenesis in cells [161]. In turn, Han et al. [67] observed higher SREBP1 expression in placental tissue and serum in patients with GDM.

GDM is associated with oxidative stress, which has implications for the mother, placental function, and foetal well-being [162]. Liu et al. [163] reported elevated miR-335-5p in endothelial cells with induction of oxidative stress and decreased SIRT7 levels in HUVECs. The authors observed that a miR-335-5p inhibitor attenuates the downregulation of SIRT7 expression induced by oxidative stress in HUVECs. In turn, SIRT7 overexpression is associated with a rescue effect against miR-335-5p-induced endothelial dysfunction. Therefore, it can be assumed that SIRT7 may play a role in the functioning of the vascular endothelium under conditions of oxidative stress and may have a potential association with GDM.

**Table 1:** Associations between sirtuin isoforms and gestational diabetes mellitus or high glucose in vitro.

Isoform	Sirtuin Expression *	Target	Principal Function	Reference
SIRT1	↓placenta	↑SERBP1 ↑NLRP3 ↑IL1β ↑IL18	lipid synthesis and insulin signalling pathways inflammation	[67]
	↑leukocytes	↑G6PD ↑IL6 ↑SNAP23	oxidative stress, inflammation, molecular transport functions	[65]
	placenta	↑miR135a5p	inflammation, adipogenesis, and glucose metabolism	[69]
	↓primary human trophoblast cells	↑CD36, FABP3, and FABP4	DHA transfer across trophoblasts, triglyceride accumulation, and fatty acid transporter expression	[73]
	↓endothelial colony-forming cells, human umbilical vein endothelial cells	↓FKBPL	angiogenesis	[64]
SIRT2	↓skeletal muscle	↓PKB, GSK3β	glucose transporter functional decline	[80]
	↓blood	MARCKS	diabetes-induced neural tube defects, mitochondrial abnormalities, and endoplasmic reticulum stress	[82]
SIRT3	↑HTR8/SVneo cells	↑AMPK/(mTOR) pathway ↓GPX4	ferroptosis and autophagy	[84]
	↓human islets cells	↑IL1β	cellular ROS accumulation apoptosis of beta cells	[93]
	↓skeletal muscle	↓MnSOD	oxidative stress	[94]
SIRT4	↓ECFCs, HUVECs	not rated	long-term cardiovascular complications	[86]
SIRT5	↓human ovarian follicular cells	↓CPS1	accumulation of ammonia in the follicular fluid	[121]
SIRT6	↓C17.2 cells	↑ acetylation of H3K56, H3K14, H3K9, and H3K27	diabetes-induced neural tube defects	[143]
SIRT7	placental tissue and serum blood	methylation of SREBP1a	cholesterol, fatty acid, and triglyceride biogenesis	[150]

* SIRT’s expression in tissue/cells under hyperglycaemia/high glucose condition; ↑, increase; ↓, decrease; SERBP1, sterol regulatory element-binding protein-1; NLRP3, NLR family pyrin domain containing 3; IL1β, interleukin 1 beta; IL18, interleukin 18; G6PD, glucose-6-phosphate dehydrogenase; IL6, interleukin 6; SNAP23; synaptosome associated protein 23; FABP3/4, fatty acid binding protein ¾; FKBPL, FK506-binding protein like; PKB, protein kinase; GSK3β, glycogen synthase kinase 3 beta; HTR8/SVneo, human trophoblast cell line; GPX4, glutathione peroxidase 4; MnSOD, manganese superoxide dismutase; ECFCs, endothelial colony-forming cells; HUVECs, human umbilical vein endothelial cells; CPS1, carbamoyl phosphate synthase 1; C17.2, neural stem cells.

## 4. Potential Pharmacological Interventions in GDM Involving SIRTs

Several pharmacological interventions have been tested for the prevention of GDM development. The most common pharmacological interventions that have been assessed are metformin or glyburide (glibenclamide) administration. However, both are generally not considered first-choice treatments, as they cross the placenta and reach the foetus. Metformin, synthetic dimethyl biguanide, has been used to treat GDM since the 1970s [164]. In pregnancy, its proven benefits include decreasing gestational weight gain and reducing foetal size; some studies have shown lower risk of caesarean delivery and rates of hypertension. It can reduce the need for insulin therapy but does not eliminate such need in many patients [165]. A recent study showed that metformin is a direct SIRT1-activating compound [166]. Moreover, it activates SIRT1 and inhibits the expression of NLRP3 inflammasome (NLRP3, ASC, caspase-1, IL-1β, and IL-18) and inflammatory cytokines (TNFα and IL-6) [167]. It was noted that therapy combining metformin and glibenclamide had a stronger effect on the attenuation of the expression of the *RAGE* gene and activation of *NRF2* and *SIRT1* genes when compared to metformin only [168]. An in vitro study of high glucose-cultured mesangial cells demonstrated that metformin alleviates oxidative stress and enhances autophagy via the AMPK/SIRT1-FoxO1 pathway [169]. Research by Cuyàs et al. [166] proposed that metformin binds to specific sites on the SIRT1 protein, including the allosteric site occupied by other sirtuin-activating compounds like resveratrol. This binding may induce conformational changes that enhance SIRT1’s deacetylase activity. Additionally, metformin’s interaction with the NAD^+^ binding site could facilitate more efficient substrate processing, further contributing to SIRT1 activation [166]. While available data suggest that metformin is a safe and effective option for women with GDM, there is limited information about its long-term impact on their children.

Due to the numerous reported health benefits of SIRT activation, research has been directed toward the discovery of SIRT activators, with activators of SIRT1 being the most well studied. One such activator is resveratrol (RSV), a small plant-derived polyphenol [170]. Recent studies have confirmed the link between molecular targets and signalling pathways of RSV and SIRT1 in GDM. In foetal endothelial colony-forming cells (ECFCs) and HUVECs isolated from GDM patients, RSV significantly enhanced SIRT1 expression and activity [171]. Yao et al. [172], using a genetic GDM mouse model that closely mimicked human GDM symptoms, showed that RSV greatly improved glucose metabolism and insulin tolerance. Moreover, they found that RV relieved GDM symptoms by enhancing AMPK activation, which in turn reduced the production and activity of glucose-6-phosphatase [172]. Another study examined the effects of oxidative stress on glucose transporters (GLUTs) and glucose uptake in the human placenta. The reduction in GLUT1 expression and glucose absorption caused by hypoxanthine/xanthine oxidase was reversed with the SIRT1 activator RSV. These findings indicate that oxidative stress suppresses GLUT1 expression and placental glucose uptake through a SIRT1-dependent mechanism [173].

Based on current scientific literature, there is no substantial evidence to suggest a direct interaction between glyburide and sirtuin proteins in diabetes. Further research would be necessary to explore any potential connections between glyburide and sirtuin activity. Additionally, in the case of GDM patients, it is known that glyburide requires careful use, as it has been linked to an increased risk of neonatal hypoglycaemia and, in some cases, macrosomia [164].

Incretin mimetics known as glucagon-like peptide-1 (GLP-1) agonists and dipeptidyl peptidase-4 (DPP-4) inhibitors, both used in diabetes treatment, have been associated with the upregulation of SIRT1. This upregulation may help limit oxidative stress and alleviate insulin resistance, contributing to better glucose control [170]. Sodium–glucose co-transporter 2 (SGLT2) inhibitors represent another class of medications commonly used in the treatment of type 2 diabetes mellitus. These drugs have a dual effect, helping to slow down the progressive decline in kidney function in individuals with diabetes. When compared to incretin mimetics or DPP-4 inhibitors, SGLT2 inhibitors lead to similar reductions in blood sugar levels while offering additional benefits in preserving kidney function and preventing negative renal outcomes; specifically, SGLT2 inhibitors create a fasting-like condition that activates both SIRT1 and AMPK, which reduces cellular stress [170].

Another class of drugs commonly used to manage diabetes is thiazolidinediones, a group of oral medications that promote the formation of fat cells and enhance fatty acid uptake. Pioglitazone is a notable drug in this category. The aim of the study conducted by Noureldein et al. [174] was to investigate the effect of fenofibrate, a medication used to lower cholesterol, alone and in combination with pioglitazone on serum SIRT1 and fetuin A levels in groups of obese patients with or without T2DM. After eight weeks of treatment with either fenofibrate alone or combined with pioglitazone, the researchers measured fasting glucose, HbA1c, serum lipids, and SIRT1 levels. All treatment groups showed an increase in SIRT1 levels and a decrease in inflammatory markers such as IL-6.

As has been noted, most antidiabetic drugs stimulate the expression of SIRT1 and hold therapeutic promise for diabetes mellitus; however, it should also be carefully considered that some of these interactions can have limitations or side effects. Sirtuins share structural similarities, making it challenging to develop modulators that selectively target specific isoforms. Non-selective modulation can lead to unintended effects on other sirtuin family members, potentially causing adverse outcomes.

## 5. Conclusions and Limitations

In summary, pregnancy is a state of high metabolic activity in which maintaining glucose homeostasis is of the utmost importance. At present, due to genetic, epigenetic, and environmental factors, maintaining this homeostasis is very difficult. These factors contribute to the development of GDM, the mechanisms of which are complex and advance over a substantial period of time. Pancreatic β-cells from women with GDM fail to compensate for a chronic fuel surfeit, leading to IR, hyperglycaemia, and an increased supply of glucose to the growing foetus. There is also evidence that obesity, low-grade chronic inflammation, gluconeogenesis, oxidative stress, and the placenta contribute to the pathology of GDM. Hence, there is a need to understand the molecular aspects of GDM more deeply. A rapidly expanding body of evidence supports that each member of the sirtuin family plays a role in regulating glucose metabolism. In fact, sirtuins play fundamental roles in trophoblast functions, foetomaternal inflammation, placental angiogenesis, and oxidative stress. They also appear to be involved in the regulation of pregnancy complications caused by maternal obesity and diabetes. We have summarised the current knowledge about the roles of sirtuin family members in the molecular mechanisms of inflammation, oxidative stress, placental dysfunction associated with GDM, and future complications. Table 1 summarises the most important; however, there is still much to explain. Further determination of the metabolic functions of sirtuins will make it easy to understand their function in each step of the pathomechanism of GDM. This information will be beneficial for the development of novel therapies that target sirtuins.

There are some major limitations in this study that could be addressed in future research. First of all, our review focused mostly on studies that presented simple associations between sirtuin expression or levels and GDM [50,61,62]. Excluding SIRT1, molecular studies elucidating the functional role of sirtuin’s isoforms in GDM pathophysiology are very limited. In our opinion, future understanding of these mechanisms will be fundamental to providing potential therapeutic interventions. Second, the GDM studies involved relatively small cohorts. This lowers the statistical power and limits the generalizability of findings. Moreover, there is a gap in current research related to sirtuins and their expression during the duration of the pregnancy. Most of the studies only present data collected at a single time point [50,61]. Longitudinal studies are necessary for a better understanding of the role of sirtuins in the development and progression of GDM. Moreover, the role of SIRTs in GDM and T2DM raises several unresolved questions. For instance, do these SIRTs function independently or work together in a synergistic manner to influence diabetes? How do they coordinate their actions within cells and tissues? While it is known that SIRTs are regulated through protein–protein interactions and microRNAs at the levels of transcription and translation, our understanding of the epigenetic mechanisms affecting sirtuins remains unclear. Interestingly, in certain scenarios, different sirtuin isoforms exert opposing effects on key enzymes. Most sirtuin isoforms (SIRT1, 2, 3, and 6) have a protective role in diabetes, while a smaller number (SIRT4 and 7) seem to have harmful effects [147]. However, their overall impact on the body remains unclear. While short-term studies indicate potential benefits, the prolonged activation or inhibition of these proteins could have unforeseen effects on metabolic processes and overall health [173].

## Data Availability

No new data were created or analyzed in this study. Data sharing is not applicable to this article.
