# Correction: Zgutka et al. Sirtuins and Their Implications in the Physiopathology of Gestational Diabetes Mellitus. *Pharmaceuticals* 2025, *18*, 41

**DOI:** 10.3390/ph18111616

**Published:** 2025-10-27

**Authors:** Katarzyna Zgutka, Marta Tkacz, Marta Grabowska, Wioletta Mikołajek-Bedner, Maciej Tarnowski

**Affiliations:** 1 Department of Physiology in Health Sciences, Faculty of Health Sciences, Pomeranian Medical University, 70-210 Szczecin, Poland; katarzyna.zgutka@pum.edu.pl (K.Z.); marta.tkacz@pum.edu.pl (M.T.); 2 Department of Histology and Developmental Biology, Faculty of Health Sciences, Pomeranian Medical University, 71-210 Szczecin, Poland; marta.grabowska@pum.edu.pl; 3 Department of Obstetrics and Gynecology, Pomeranian Medical University in Szczecin, 70-204 Szczecin, Poland

**References**

In the original publication [1], the reference [107] was retracted and has been replaced with the new reference as below:

107. Dai, Y.; Liu, S.; Li, J.; Li, J.; Lan, Y.; Nie, H.; Zuo, Y. SIRT4 suppresses the inflammatory response and oxidative stress in osteoarthritis. *Am. J. Transl. Res*. **2020**, *12*, 1965–1975.

**Text Correction**

In the original publication [1], the text statement corresponding to reference 107 was deleted accordingly: “, as there is a reduction in SIRT4 expression at the early stage of T2DM development when IR appears. SIRT4 overexpression downregulates the expression of the apoptosis-related proteins NADPH oxidase 1 (NOX1), Bax, and phosphorylated p38 and upregulates the expression of Bcl-2 in glucose-stimulated podocytes [107]”.

The authors state that the scientific conclusions are unaffected. This correction was approved by the Academic Editor. The original publication has also been updated.
